# Detection of Hepatic Drug Metabolite-Specific T-Cell Responses Using a Human Hepatocyte, Immune Cell Coculture System

**DOI:** 10.1021/acs.chemrestox.2c00343

**Published:** 2023-02-22

**Authors:** Serat-E Ali, Xiaoli Meng, Laila Kafu, Sean Hammond, Qing Zhao, Monday Ogese, Rowena Sison-Young, Robert Jones, Benjamin Chan, Lucia Livoti, Yonghu Sun, Lele Sun, Hong Liu, Anthony Topping, Christopher Goldring, Furen Zhang, Dean John Naisbitt

**Affiliations:** † Department of Molecular and Clinical Pharmacology, University of Liverpool, Ashton Street, Liverpool L69 3GE, U.K.; ‡ Proteintech Group, 4th Floor, 196 Deansgate, Manchester M3 3WF, U.K.; § Apconix Alderley Park, Alderley Edge, Cheshire SK10 4TG, U.K.; ∥ Shandong Provincial Hospital for Skin Diseases & Shandong Provincial Institute of Dermatology and Venereology, Shandong First Medical University & Shandong Academy of Medical Sciences, Jinan, Shandong, China; ⊥ Department of Hepatobiliary Surgery, Aintree University Hospital, Liverpool University Hospitals, NHS Foundation Trust, Liverpool L9 7AL, U.K.; # School of Engineering, The Quadrangle, The University of Liverpool, Brownlow Hill, Liverpool L69 3GH, U.K.

## Abstract

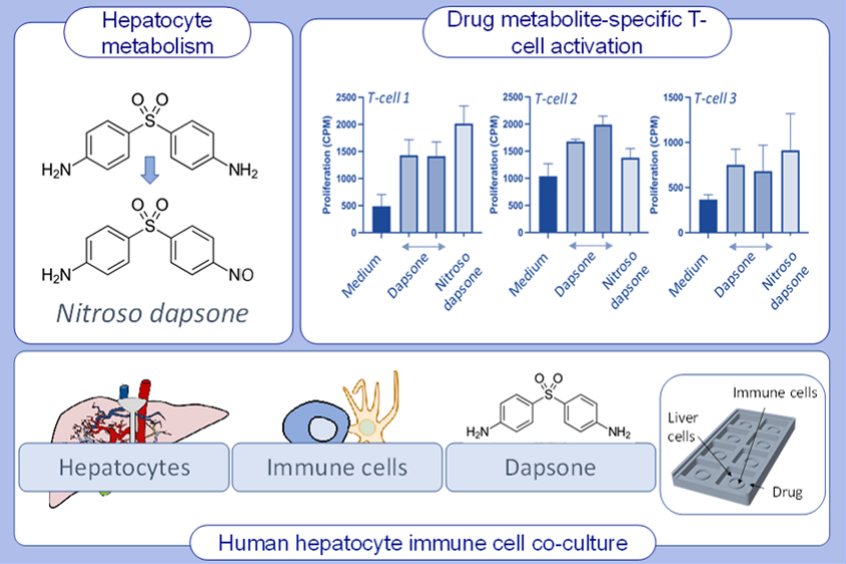

Drug-responsive T-cells are activated with the parent compound or metabolites, often via different pathways (pharmacological interaction and hapten). An obstacle to the investigation of drug hypersensitivity is the scarcity of reactive metabolites for functional studies and the absence of coculture systems to generate metabolites in situ. Thus, the aim of this study was to utilize dapsone metabolite-responsive T-cells from hypersensitive patients, alongside primary human hepatocytes to drive metabolite formation, and subsequent drug-specific T-cell responses. Nitroso dapsone-responsive T-cell clones were generated from hypersensitive patients and characterized in terms of cross-reactivity and pathways of T-cell activation. Primary human hepatocytes, antigen-presenting cells, and T-cell cocultures were established in various formats with the liver and immune cells separated to avoid cell contact. Cultures were exposed to dapsone, and metabolite formation and T-cell activation were measured by LC–MS and proliferation assessment, respectively. Nitroso dapsone-responsive CD4+ T-cell clones from hypersensitive patients were found to proliferate and secrete cytokines in a dose-dependent manner when exposed to the drug metabolite. Clones were activated with nitroso dapsone-pulsed antigen-presenting cells, while fixation of antigen-presenting cells or omission of antigen-presenting cells from the assay abrogated the nitroso dapsone-specific T-cell response. Importantly, clones displayed no cross-reactivity with the parent drug. Nitroso dapsone glutathione conjugates were detected in the supernatant of hepatocyte immune cell cocultures, indicating that hepatocyte-derived metabolites are formed and transferred to the immune cell compartment. Similarly, nitroso dapsone-responsive clones were stimulated to proliferate with dapsone, when hepatocytes were added to the coculture system. Collectively, our study demonstrates the use of hepatocyte immune cell coculture systems to detect in situ metabolite formation and metabolite-specific T-cell responses. Similar systems should be used in future diagnostic and predictive assays to detect metabolite-specific T-cell responses when synthetic metabolites are not available.

## Introduction

Drug hypersensitivity reactions remain a major obstacle in the development of safe therapeutics. T-lymphocytes are believed to be the ultimate mediators of the adverse reaction with an human leukocyte antigen (HLA)-drug-peptide binding interaction being the molecular initiating event.^1^ T-cells are activated via two independent pathways with (i) the drug binding directly to the HLA or HLA-associated peptides via a reversible interaction (pharmacological interaction [PI] concept)^2−5^ or (ii) the drug undergoing metabolic activation, with the derived metabolites forming covalently bound protein adducts.^6−8^ Protein adducts are processed by antigen-presenting cells and the derived drug-modified peptides are thought to associate with HLA proteins to stimulate T-cells.^9−12^ The direct binding interaction of drugs to HLA and subsequent T-cell activation is relatively easy to study through molecular modeling, structural analyses, and functional studies with commercially available drugs and peripheral blood mononuclear cells (PBMCs) from hypersensitive patients, and significant progress has been made linking specific HLA associations detected in genome-wide association studies to selective drug HLA binding and T-cell activation.^4−6,13^ In contrast, assessment of drug metabolite T-cell responses are much more difficult to investigate since (i) with the exception of sulfa drugs (e.g., sulfamethoxazole and dapsone), synthetic drug metabolites are not available for functional studies,^14,15^ (ii) human PBMC culture systems do not express metabolizing enzymes to generate reactive intermediates in the quantity required to activate T-cells,^6,8^ and (iii) metabolite generating cell–immune cell coculture systems are difficult to establish due to allogeneic T-cell activation. Therefore, in this study, we utilized dapsone metabolite-responsive T-cells from patients with hypersensitivity, alongside a primary human hepatocyte-metabolizing system, separated through novel cell culture plates, to drive in situ metabolite formation and subsequent T-cell activation. To establish the system, dapsone metabolite-responsive T-cell clones, which displayed no cross-reactivity with the parent drug, were isolated and characterized using defined protocols. In situ hepatocyte metabolite formation was analyzed by mass spectrometry.

Dapsone is a sulfa (SO_2_) antibiotic, containing two para-amino-substituted aromatic rings, which is used for the treatment of opportunistic infections.^16,17^ Dapsone hydroxylamine, an oxidative metabolite of dapsone, is formed by hepatic and extra-hepatic (e.g., cytochrome P450, cyclooxygenase, and peroxidase) metabolism. The hydroxylamine undergoes auto-oxidation to generate nitroso dapsone, a haptenic compound that binds covalently to cysteine residues on protein.^15,18−22^ Although dapsone is an effective therapeutic agent, widely used for the treatment of leprosy, its use is often overshadowed by the development of a hypersensitivity syndrome that manifests as fever, papular or exfoliative rash, hepatitis, and generalized lymphadenopathy. The prevalence of such adverse events is ∼1.4% with around 10% resulting in death.^23^ The development of dapsone hypersensitivity in Thai, Chinese, and Taiwanese populations is strongly associated with HLA-B*13:01 expression;^24−27^ patients display positive results following skin testing, and PBMCs are activated with both dapsone and the nitroso metabolite.^6,23,26^ Importantly, dapsone and nitroso dapsone activate CD4+ and CD8+ T-cells in all hypersensitive patients via different pathways, PI and hapten, respectively.^6,28^ Although certain T-cells display cross-reactivity and are activated in the presence of dapsone and nitroso dapsone, others do not. They are highly specific and activated with either the parent drug or nitroso metabolite. The availability of nitroso dapsone T-cell clones that do not cross-react with the parent drug provide the ideal resource to explore whether dapsone metabolites generated through hepatic metabolism form protein adducts in situ in quantities that are sufficient to stimulate a T-cell response. Three different coculture approaches are described that combine primary human hepatocytes with EBV-transformed B-cells and cloned T-cells from hypersensitive patients, an IdMOC coculture system, a transwell system, and a self-designed 3D-printed system, to explore the optimal culture requirements.

## Experimental Procedures

### Generation of Nitroso Dapsone-Responsive T-Cell Clones

PBMCs were isolated using density gradient separation with Lymphoprep (Axis Shield, Dundee, UK) from a dapsone-hypersensitive patient for the generation of a panel of nitroso dapsone-responsive T-cell clones. Consent was provided and the study was approved by the Ethical Committee of the Shandong Provincial Institute of Dermatology and Venereology. A material transfer agreement was signed prior to shipment of PBMCs to the University of Liverpool. PBMCs (2 × 10^6^ cells/well; 1 mL) were cultured in the presence of nitroso dapsone (20–40 μM) for 14 days in R9 medium (RPMI 1640 supplemented with 10% human AB serum (Class A; Innovative Research Inc., Novi, MI), 25 mM HEPES, 10 mM l-glutamine, and 25 mg/mL transferrin (Sigma-Aldrich, Gillingham, UK)). On day 6 and 9, cultures were stimulated with 200 IU/mL recombinant human interleukin (IL-2) (PeproTech, London, UK). To generate and expand drug-specific clones, a serial dilution and repetitive mitogen expansion method was employed as previously described.^24^

After expansion, T-cells (5 × 10^4^) and autologous irradiated Epstein–Barr virus (EBV)-transformed B-cells (1 × 10^4^) were incubated with nitroso dapsone (40 μM) in a 96-well U-bottomed plate for 48 h to test for drug specificity. [^3^H] thymidine (0.5 μCi/well, 5 Ci/mmol; Moravek Biochemicals, Brea, CA) was added for the final 16 h to measure proliferation in drug-treated wells in comparison to media control. Clones demonstrating a stimulation index (SI) (proliferation in the presence of compound/proliferation in control wells) of 2 or above were considered drug-responsive and expanded for further characterization.

To generate EBV-transformed B-cell lines, filtered supernatant from the EBV-producing cell line (B95.8) was cultured with PBMCs alongside cyclosporine A (1 μg/mL; Sigma-Aldrich). The resulting EBV-transformed B-cells then served as an immortalized source of autologous antigen-presenting cells, which were sustained in maintenance media (RPMI 1640 supplemented with 10% fetal bovine serum (Invitrogen, Paisley, UK), 100 mM l-glutamine, penicillin, and streptomycin).

### Cross-Reactivity of T-Cell Clones

To demonstrate drug-dose dependency of T-cell clones, T-cells (5 × 10^4^ cells per well) were cultured with irradiated autologous EBV-transformed B-cells (1 × 10^4^ cells per well) alongside increasing nitroso dapsone (0–40 μM) concentrations. Two concentrations of previously optimized concentrations of the parent compound^6^ were also included to identify any cross-reactive T-cell clones. Clones demonstrating dose dependency as well as no cross-reactivity with the parent compound were expanded and utilized for further characterization experiments.

### Cytokine and Cytolytic Molecule Release Profiles

The enzyme-linked immunospot (ELISpot) assay was employed to evaluate secretory molecules (IFN-γ, granzyme B, IL-5, perforin, IL-13, IL-17, IL-22, and Fas-L) released by clones upon challenge with the parent drug and metabolite. Plates were precoated with the target capture antibody for 24 h, cocultures (5 × 10^4^ T-cell clones and 1 × 10^4^ irradiated autologous EBV-transformed B-cells) were incubated in the presence of media, dapsone, or nitroso dapsone at various concentrations for 48 h. Plates were subsequently washed, a secondary biotin antibody was added, and secreted molecules were visualized using an AID ELISpot reader (Oxford Biosystems Cadama, Oxfordshire, UK) in line with manufacturer’s instructions (Mabtec).

### Assessment of Pathways of T-Cell Activation

To determine pathways of drug presentation, glutaraldehyde fixation (0.05%; Sigma-Aldrich) was utilized to inhibit intracellular processes in EBV-transformed B-cells associated with antigen processing. Simultaneously, EBV-transformed B-cells were pulsed (24 h) with optimal concentrations of nitroso dapsone followed by washing to remove unbound drugs. T-cell clones were then cultured in the presence/absence of unpulsed, pulsed, and glutaraldehyde-fixed EBV-transformed B-cells for 48 h, followed by addition of [^3^H] thymidine (0.5 μCi/well, 5 Ci/mmol) for the final 16 h of incubation to measure proliferative responses.

### Viability Assessment of PHH Using the CellTiter-Glo Cell Viability Assay

To assess hepatocyte toxicity of dapsone, primary human hepatocytes were plated in collagen-coated, flat-bottomed 96-well plates (Thermo Scientific) and incubated with various concentrations of dapsone for 24 h at 37 °C, 5% CO_2_. The viability of the cells was then analyzed using the CellTiter-Glo Cell Viability Assay, in comparison to untreated controls.

### Establishment of a T-Cell Clone, Antigen-Presenting Cell, and Primary Human Hepatocyte Coculture System

For the development of a coculture model, primary human hepatocytes were isolated from liver biopsies collected from liver resections of varying etiologies conducted at the University of Liverpool Teaching Hospital (Aintree, Liverpool). Written informed consent was obtained from donors to partake in the research study, approved by the local Liverpool research ethics committee. Biopsies were initially perfused with warm (37 °C) 1× HEPES to remove residual blood for 20–40 min, depending on sample size. Following the perfusion phase, collagenase type IV (Sigma-Aldrich) was utilized to digest tissue. Upon successful digestion, the liver biopsy was cut open to release encapsulated hepatocytes. Hepatocytes were collected into William’s E medium and washed twice using gradient centrifugation (5 min, 4 °C, and 80 *g*) and counted using trypan blue (Sigma-Aldrich) staining and light microscopy. Hepatocytes were then cultured in William’s E media supplemented with l-glutamine (2 mM), penicillin (100 μg/mL), streptomycin (100 U/mL), insulin-transferrin-selenium (100×), and dexamethasone (1 μM/mL) on either precoated collagen plates (Corning, Flintshire, UK), rat tail collagen type IV coated (Invitrogen) in IdMOC (Merck), or in-house (developed at the University of Liverpool) coculture plates at a density of 150–250,000 cells per cm^2^. To ensure cells were adequately seeded, they were assessed and imaged using a light microscope. Primary hepatocytes were then cultured with drug-specific T-cell clones, antigen-presenting cells, and the drug.

For T-cell experimentation, three assays were used: an IdMOC coculture system, a transwell system, and a self-designed 3D-printed system. Since clones are generated from a single patient precursor cell, they can only be generated in a finite number. For this reason, only clones displaying the same specificity (nitroso dapsone-specific) and activated via a hapten pathway were used in the coculture assays.

(i) IdMOC plates: 24 h after hepatocyte seeding to 2/6 wells in each IdMOC chamber, T-cells were harvested, washed, and diluted to 5 × 10^6^ cells/mL in the hepatocyte medium without dexamethasone. Irradiated autologous EBV-transformed B-cells were harvested and diluted to 1 × 10^6^, mixing them directly into the T-cell suspension. Fifty microliters of T-cell/antigen-presenting cell coculture was plated into 4/6 wells of the IdMOC chambers (wells where no hepatocytes were plated). The plates were then returned to an incubator for 1 h to allow T-cells to settle in the well. The individual chambers were then carefully loaded with 1.5 mL of dapsone. Two wells were used as negative (medium only) and positive controls (nitroso dapsone). The plate was then returned to an incubator for 24 h at 37 °C, 5% CO_2_. After 24 h, the supernatants were carefully removed, and T-cell wells were carefully transferred into U-bottom 96 well plates. These plates were incubated for a further 24 h at 37 °C, 5% CO_2_, and proliferation was assessed through the addition of [3H]-thymidine for the final 16 h as described above. Proliferative responses were compared to the negative and positive controls to assess hepatocyte metabolite-derived T-cell proliferative responses. The supernatant from the coculture was frozen immediately for mass spectrometry analysis.
(ii) 24-well plate assay: 24 h after hepatocyte seeding, T-cells were harvested, washed, and diluted to 2.5 × 10^6^ cells/mL in the hepatocyte medium (without dexamethasone). Irradiated autologous EBV-transformed B-cells were harvested and diluted to 0.5 × 10^6^, mixing directly into the T-cell suspension. The hepatocyte medium was then removed, and inserts (1 μM) (Millicell Hanging Cell Culture Insert PET 1 μm, 24-well, Merck) were placed above the hepatocytes within the 24-well plate. Two hundred microliters of T-cell/antigen-presenting cell suspension was transferred into individual insert upper chambers. The chamber and well were then filled with dapsone, negative (medium) or positive control (nitroso dapsone). The plate was returned to an incubator for 48 h at 37 °C, 5% CO_2_. After 24 h, the supernatants were carefully removed, and T-cell wells were carefully transferred into U-bottom 96 well plates. These plates were incubated for a further 24 h at 37 °C, 5% CO_2_, and proliferation was assessed through the addition of [3H]-thymidine for the final 16 h as described above. EBV-transformed B-cells and T-cell clones were placed in the top compartment with the assumption that hepatocyte-derived metabolites will diffuse in the media to expose the B- and T-cells. Mass spectrometry studies described below were performed to confirm that the metabolites formed do circulate in media (albeit we cannot confirm whether exposure to all cells was homogeneous).
(iii) Self-designed 3D-printed system: 24 h after hepatocyte seeding into each outer well, T-cells were harvested, washed, and diluted to 2.5 × 10^6^ cells/mL in the hepatocyte medium (without dexamethasone). Irradiated autologous EBV-transformed B-cells were harvested and diluted to 0.5 × 10^6^ cells and mixed directly into the T-cell suspension. The overlaying medium was removed from the wells and the T-cell/APC coculture was plated into the central well without disturbing the hepatocytes. The fresh hepatocyte medium (without dexamethasone) was then placed to cover the hepatocytes. The plates were then incubated for 30 min–1 h to allow immune cells to settle into the well. The individual chambers were then topped with 1.5 mL of hepatocyte medium, dapsone, or nitroso dapsone (positive control). The plate was then returned to an incubator for 48 h at 37 °C, 5% CO_2_. After 48 h, the supernatants were carefully removed, and T-cell wells were carefully transferred into U-bottom 96 well plates. These plates were incubated for a further 24 h at 37 °C, 5% CO_2_, and proliferation was assessed through the addition of [3H]-thymidine for the final 16 h.

### Glutathione Trapping of the Formed Dapsone Metabolites

Glutathione trapping experiments were conducted via addition of glutathione to hepatocytes, immune cells, and parent compound cocultures. Four volumes of 100% acetonitrile was added to the supernatant to stop metabolism before centrifugation and drying using a speedvac concentrator (Eppendorf, Hamburg, Germany). Samples were then reconstituted in 20 μL of acetonitrile and 80 μL of LC–MS ultrapure water (Sigma-Aldrich). Multiscreen filter plates (Sigma-Aldrich), prewet with 200 μL of water, were utilized to filter the samples using the centrifugation process as per manufacturer’s instruction. Fifty microliters of the sample was then transferred to HPLC vials (VWR, Radnor, PA, USA) for metabolite identification using a QTRAP 5500 mass spectrometer (AB Sciex), coupled with a Dionex UltiMate 3000 HPLC system (Thermo Fisher) and Kinetex C18 column (2.6 μm C18, 100 mm × 2.1 mm, Phenomenex, Macclesfield, Cheshire, UK). Mobile phase A was 0.1% formic acid and B was acetonitrile with 0.1% formic acid (v/v). The gradient was: 0–6 min 70% B, 7–10 min 95% B, and 10.1–16 min 5% B. The flow rate was 200 μL/min. The MS/MS experiments were performed using either multiple reaction monitoring (MRM) or precursor ion scanning of 156.01, a characteristic fragment from dapsone. The MRM transitions were selected as follows: dapsone, 249/156, dapsone hydroxylamine, 265/108, mono-acetyl dapsone, 290/249, and dapsone glutathione, 602.1/156 and 586.1/156. MRM survey scans were used to trigger enhanced product ion MS/MS scans of analytes, with Q1 set to unit resolution and dynamic fill selected. Data were analyzed using Analyst software, version 1.5.1 (AB Sciex).

## Results

### Isolation and Characterization of Nitroso Dapsone-Specific T-Cell Clones

**Figure 1:**
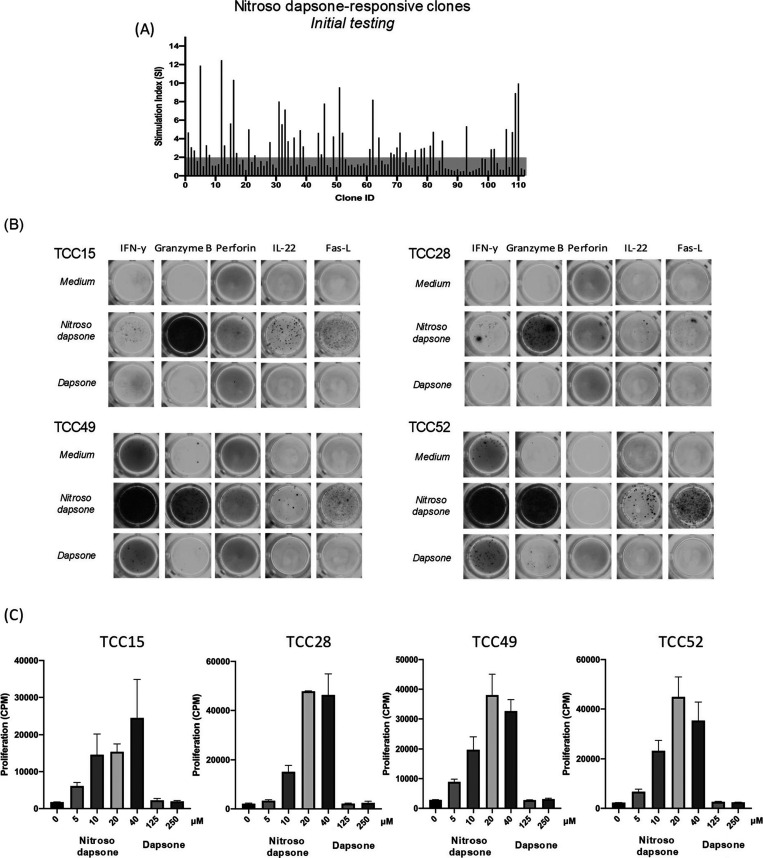
Generation of nitroso dapsone-specific clones and assessment of cross-reactivity. (A) PBMCs from a patient with dapsone hypersensitivity were cultured with nitroso dapsone (20–40 μM) for 14 days. Clones were then generated through serial dilution and repeated mitogen stimulation. Clones (0.5 × 10^5^) were cultured with autologous EBV-transformed B-cells (0.1 × 10^5^) and DDS-NO (20 μM) in duplicate for 48 h at 37 °C, 5% CO_2_. For the final 16 h of culture, [3H]-thymidine (0.5 μCi/well) was added to assess proliferation. TCCs demonstrating an SI > 2 were expanded for further characterization. (B) ELISpot was used for the detection of IFN-γ, GB, IL-5, perforin, IL-13, IL-17, IL-22, and Fas-L from clones exposed to nitroso dapsone. Drug-specific TCCs (0.5 × 10^5^) were cultured with autologous EBV-transformed B-cells (0.1 × 10^5^) and dapsone or nitroso dapsone in ELISpot plates precoated with the target cytokine antibody for 48 h. Plates were then processed and developed using the relative secondary Abs and the secretion was visualized as spots. PHA was used as a positive control. (C) To identify metabolite-specific T-cell clones which do not respond to the parent drug, clones were cultured with increasing concentration of dapsone and nitroso dapsone in the presence of autologous EBV-transformed B-cells for 48 h at 37 °C, 5% CO_2_. [3H]-thymidine was added for the final 16 h to measure proliferation.

**Figure 2:**
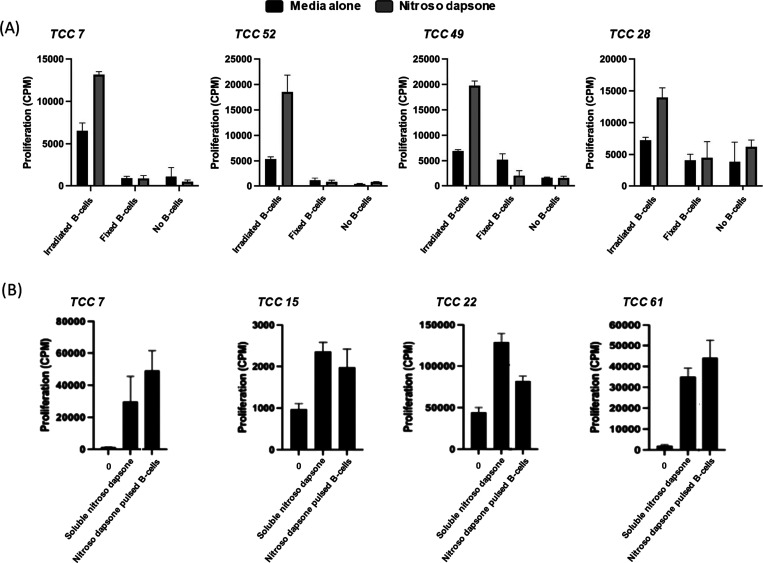
Pathway of activation for nitroso-dapsone-specific T-cell clones. (A) Drug-responsive T-cells were cultured for 48 h with nitroso dapsone and glutaraldehyde-fixed or irradiated EBV-transformed B-cells, or in the absence of B-cells. [3H]-thymidine was added for the final 16 h for proliferative assessments. (B) Autologous EBV-transformed B-cells were pulsed with nitroso dapsone for 24 h before washing to remove the unbound drug. These B-cells were then cultured with clones alongside in the absence of soluble drugs for 48 h. Soluble nitroso dapsone served as a positive control. [3H]-thymidine was added for the final 16 h to measure proliferative responses.

One hundred and twelve T-cell clones were generated from hypersensitive patient nitroso dapsone PBMC cultures. Initial testing of clones (in the presence of antigen-presenting cells) identified that 30 were stimulated to proliferate in the presence of nitroso dapsone (Figure 1A) These nitroso dapsone-responsive clones were expanded and tested for proliferative responses against increasing drug metabolite concentrations. A vast majority (29/30) of clones were stimulated to proliferate in a dose-dependent manner and secrete varying levels of IFN-γ, IL-22, granzyme B, and Fas-L in response to 40 μM nitroso dapsone (Figure 1B,C); therefore, these clones were used for further experimentation. Detection of different levels of cytokines with individual clones is consistent with our previous study characterizing the phenotype of nitroso dapsone-responsive T-cell clones.^6^ For the clones to be used in the hepatocyte coculture, it was critical to show that they are not activated with dapsone, the parent drug. Of the 29 nitroso dapsone-responsive T-cell clones, nine clones were found to display no reactivity against dapsone at concentrations up to 250 μM in proliferation and cytokine release assays (Figure 1B,C; four representative clones shown). To elucidate the effect of antigen processing on nitroso dapsone-specific T-cell proliferation, antigen-presenting cells were fixed with glutaraldehyde prior to drug metabolite exposure. Upon antigen-presenting cell fixation, proliferative responses of clones with nitroso dapsone were diminished. A similar effect was also observed when antigen-presenting cells were removed from the assay (Figure 2A). Since nitroso dapsone binds covalently to cysteine residues on cellular protein, antigen-presenting cells were pulsed with the drug metabolite for 24 h. The pulsed antigen-presenting cells were washed to remove the unbound drug metabolite and added to the T-cell clones, and proliferative responses were measured. Clones were stimulated to proliferate with antigen-presenting cells pulsed with nitroso dapsone (Figure 2B). Collectively these data characterize a panel of nitroso dapsone-specific T-cell clones activated via a pathway dependent on formation for protein adducts and protein processing by antigen-presenting cells, which can be used as a readout to establish a hepatocyte immune cell coculture system to measure drug metabolite-specific T-cell responses where only parent drug is available for functional studies. All of the nine clones expressed the CD4+ coreceptor.

### Dapsone Does Not Induce Direct Hepatocyte Toxicity

**Figure 3:**
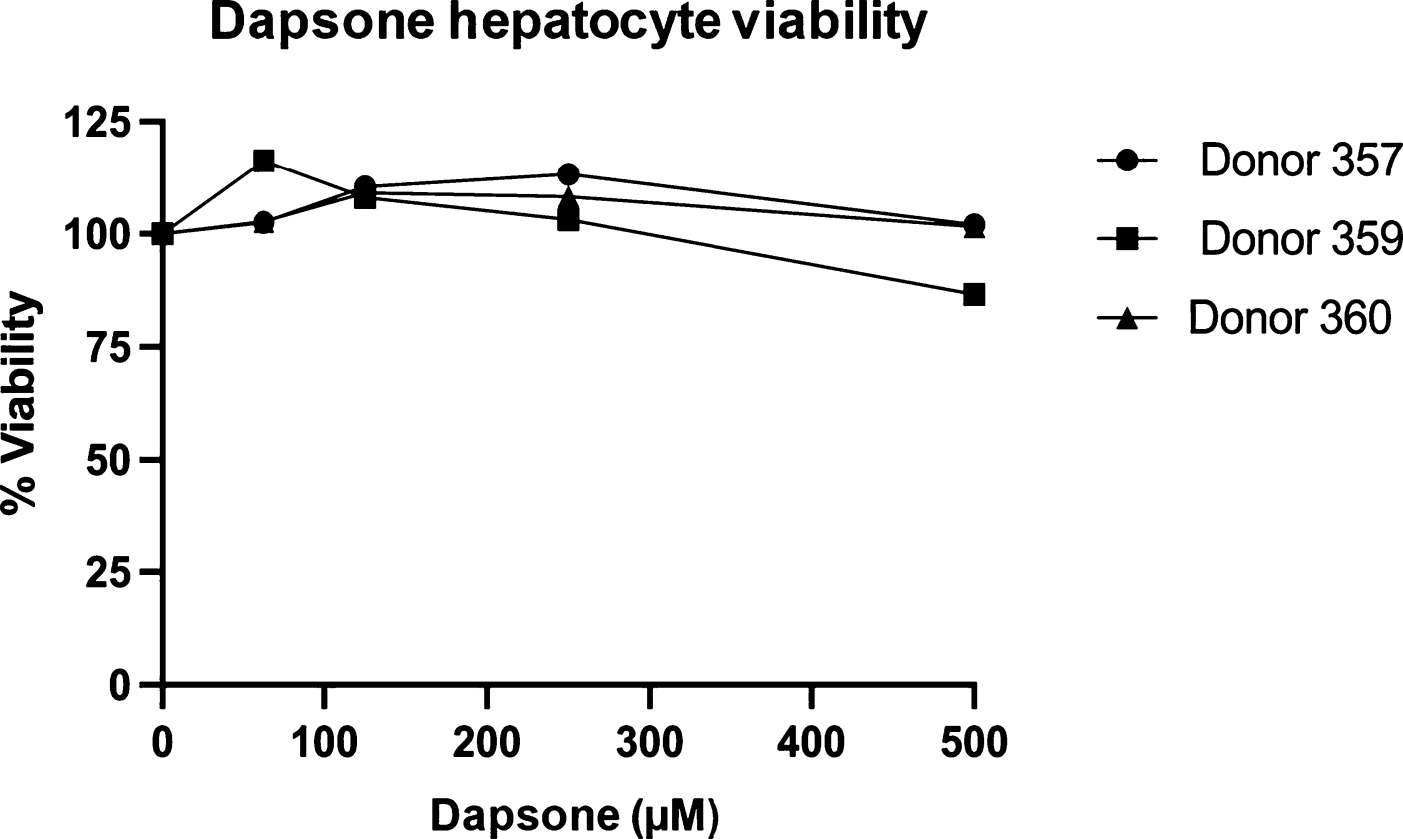
Viability of primary human hepatocytes upon dapsone exposure. To assess whether the drug concentrations utilized in the coculture were nontoxic, primary human hepatocytes were cultured in the presence of increasing concentrations of dapsone for 48 h. The CellTiter-Glo Cell Viability Assay, a luminescence-based assay, was used to measure viability of hepatocytes. Blank and untreated control wells were used to calculate a % viability value (i.e., % of mean viable cells in comparison to the untreated control once adjusted for background luminescence). The experiment was repeated on separate occasions with hepatocytes from three donors.

To ensure dapsone did not diminish the viability of human hepatocytes, cells were exposed to increasing concentrations of the parent compound and toxicity assessed. No reduction in hepatocyte viability was observed with dapsone at the concentrations used in the coculture assays (Figure 3).

### IdMOC Plates Allow for Culture of Hepatocytes with Drug-Specific T-Cell Clones, but Hepatocyte-Derived Dapsone Metabolite-Specific T-Cell Responses Are Not Detected

**Figure 4:**
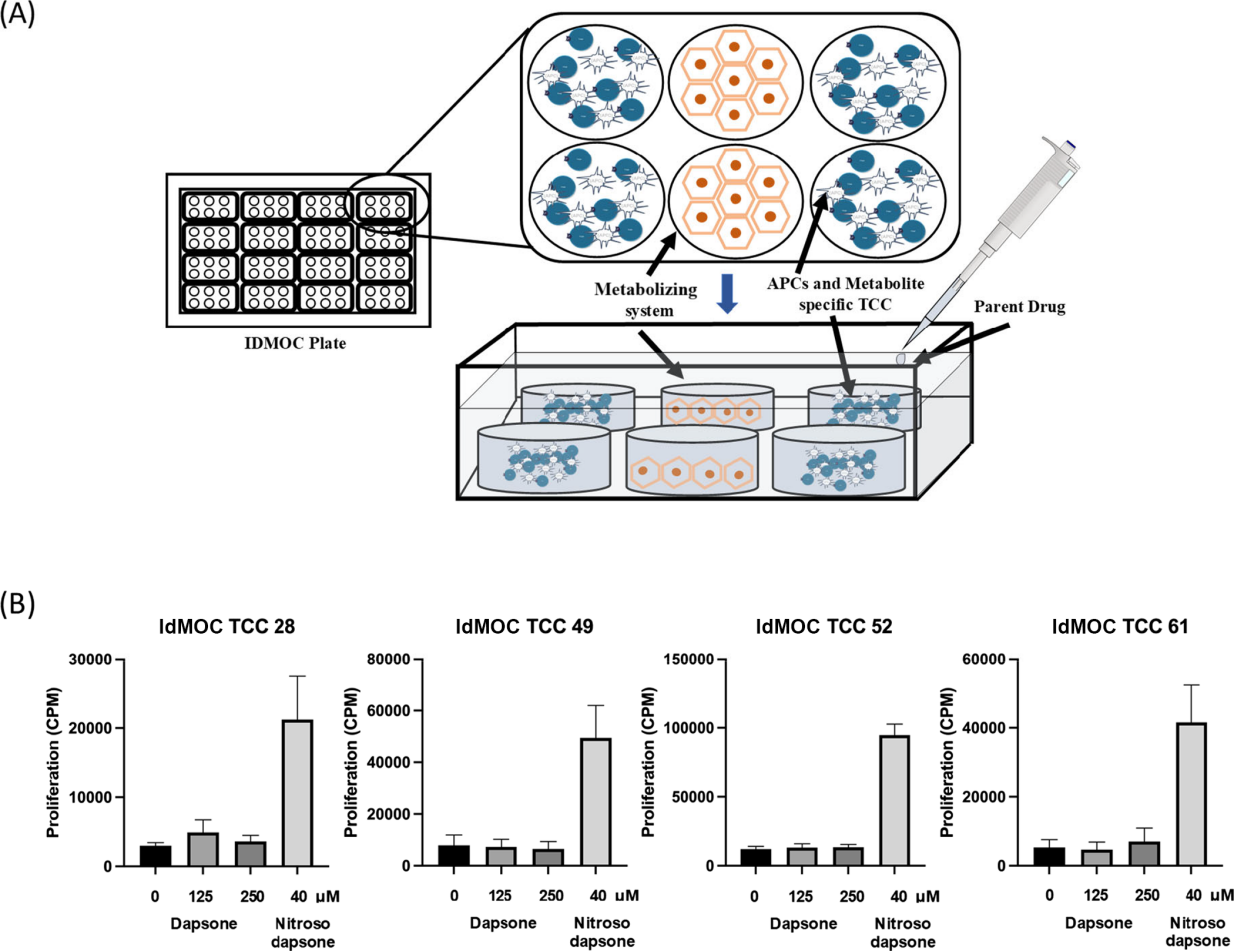
Establishment of the IdMOC hepatocyte immune cell coculture system for metabolite generation and T-cell activation studies. (A) Freshly isolated primary human hepatocytes were plated in the middle two wells of a 96-well chamber with T-cells and EBV-transformed B-cells plated in the outer four wells. The chamber was then loaded with dapsone, covering all cellular components. (i) Seeded hepatocytes after 48 h culture and (ii) undisturbed clones and EBV-transformed B-cells after 48 h culture. Similar chambers were set up with medium only and nitroso dapsone as negative and positive controls (B) after the initial culture with hepatocytes and T-cell clones were moved to 96-well U-bottomed plates and cultured for a further 24 h. [3H]-thymidine was used to measure proliferative responses.

To measure metabolite generation and subsequent T-cell activation, IdMOC plates were initially utilized. Hepatocytes were seeded alongside EBV-transformed B-cells and nitroso dapsone-specific T-cell clones, in separate compartments (Figure 4A), and covered with an overlaying medium containing dapsone. To assess whether clones cultured alongside antigen-presenting cells and hepatocytes proliferate upon parent drug exposure, T-cells were harvested after the culture period and proliferation was measured with [3H]-thymidine. Upon culture of T-cell clones with dapsone, no significant increase in proliferation was observed compared to the media controls (Figure 3B). However, in all chambers exposed to the positive control nitroso dapsone, a significant proliferative response was seen (Figure 4B).

### Use of Multiwell Inserts and Self-Designed 3D-Printed (Liv-3D) Plates for Dapsone Metabolite Generation and Assessment of In Situ Hepatocyte-Derived, Metabolite-Specific T-Cell Responses

**Figure 5:**
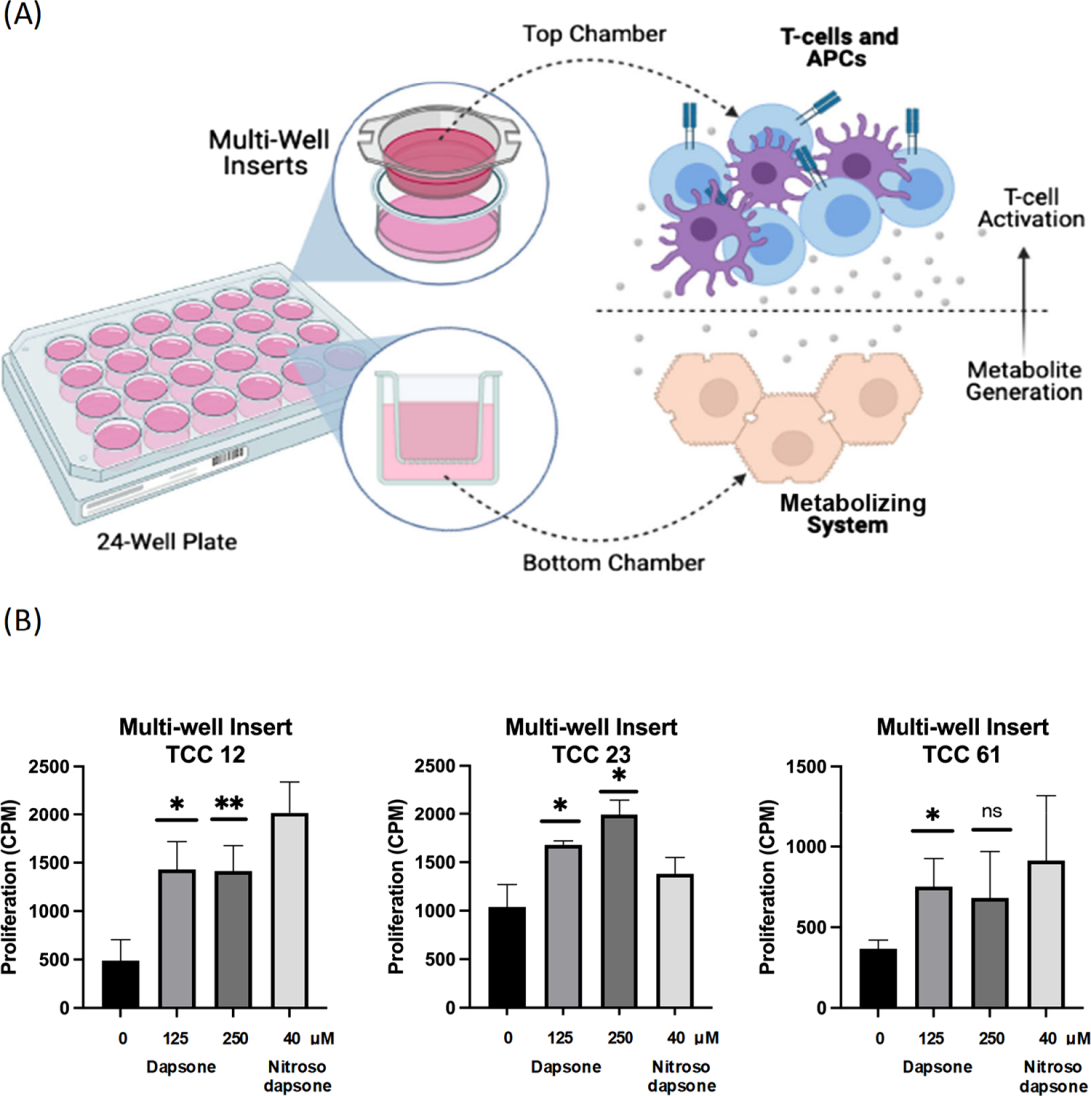
Establishment of a transwell hepatocyte immune cell coculture system for metabolite generation and T-cell activation studies. (A) Primary human hepatocytes were plated in collagen-coated 24-well plates. An insert was fitted on top of the hepatocyte wells and loaded with T-cell clones and EBV-transformed B-cells. Individual wells were then loaded with an overlaying medium containing medium control, dapsone, or nitroso dapsone (as a positive control). (B) After 48 h, T-cells were harvested from the insert, transferred to 96-well U-bottomed plates, and cultured for a further 24 h. [3H]-thymidine was then added to assess proliferative responses. Statistical analysis compares cultures with and without drugs, Student’s *t-*test (*P < 0.05; ** *P* < 0.01).

**Figure 6:**
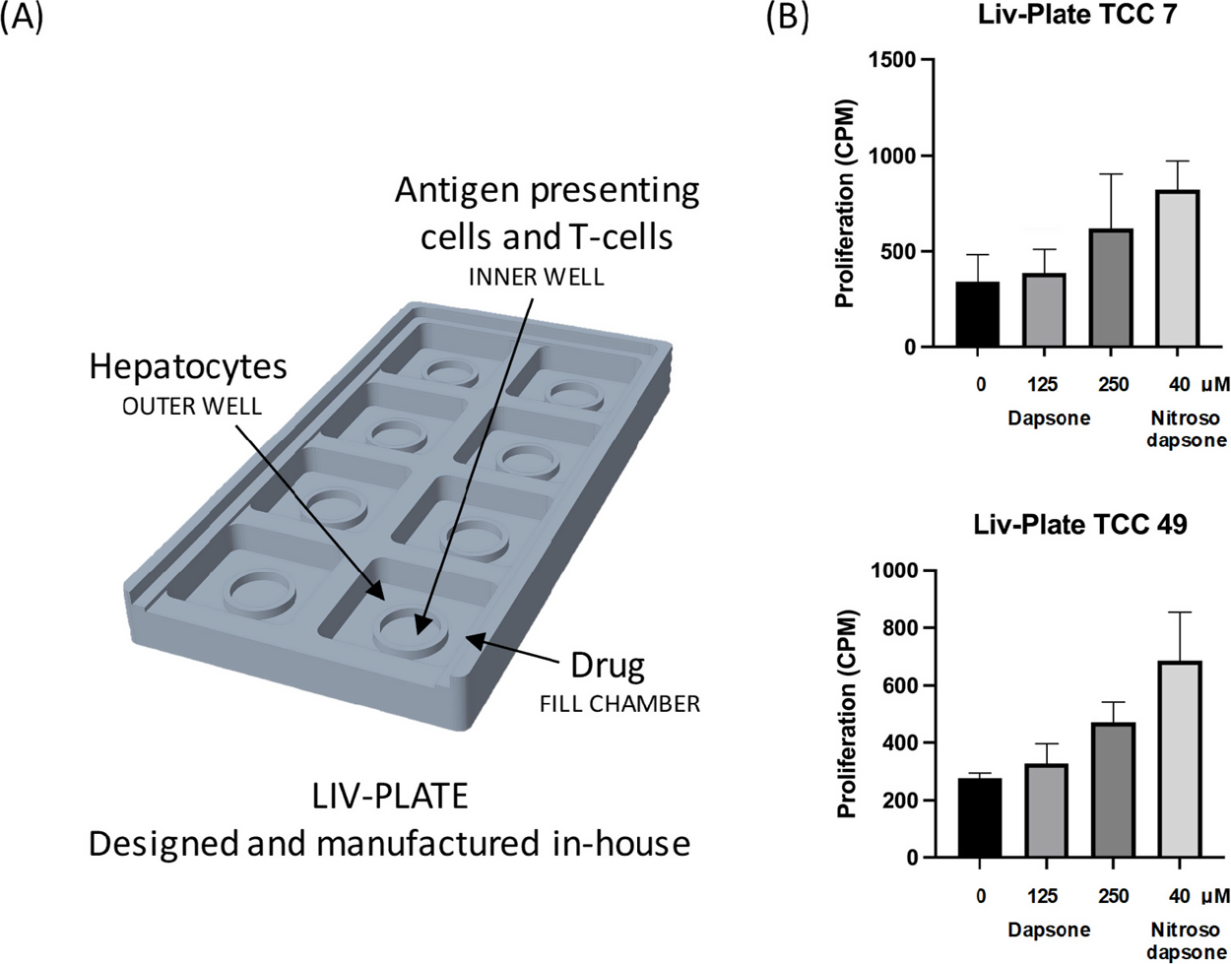
Establishment of an in-house designed and printed hepatocyte immune cell coculture system for metabolite generation and T-cell activation studies. (A) Self-designed plate has a larger surface area to seed hepatocytes and immune cells. Clones and EBV-transformed B-cells were separated from hepatocytes as indicated. Medium, dapsone, or nitroso dapsone (positive control) were added to the cells for 48 h. T-cells were then harvested, transferred to 96-well U-bottomed plates, and cultured for a further 24 h. [3H]-thymidine was then added to assess proliferative responses.

To assess the utility of multiwell inserts in a metabolite generating system, hepatocytes were seeded in 24-well plates and then cultured with a multiwell insert lined with nitroso dapsone-responsive T-cell clones and antigen-presenting cells. This layout allows for hepatocytes and T-cell clones to be in proximity without physical contact but with a permeable membrane for transfer of small molecules (Figure 5A). Upon culture of clones in the dapsone metabolite generating system, three out of four showed an increase in proliferation when compared with the medium control (Figure 5B). Similarly, utilizing an in-house designed and 3D-printed plate (Liv-Plate) with a novel layout (Figure 6A), clones were cultured alongside hepatocytes and antigen-presenting cells. After coculture, clones were transferred into U-bottom 96 well plates and left to incubate for a further 24 h. Two out of three dapsone-treated clones were stimulated to proliferate (Figure 6B).

T-cell clones exposed to the positive control, nitroso dapsone, in multiwell inserts and Liv-3D plates were found to proliferate to a lower extent when compared to the same clones cultured under classical conditions (i.e., clones, irradiated B-cells, and nitroso dapsone in the absence of hepatocytes in a 96-well round-bottomed plate).

### Detection of Nitroso Dapsone Glutathione Adducts

**Figure 7:**
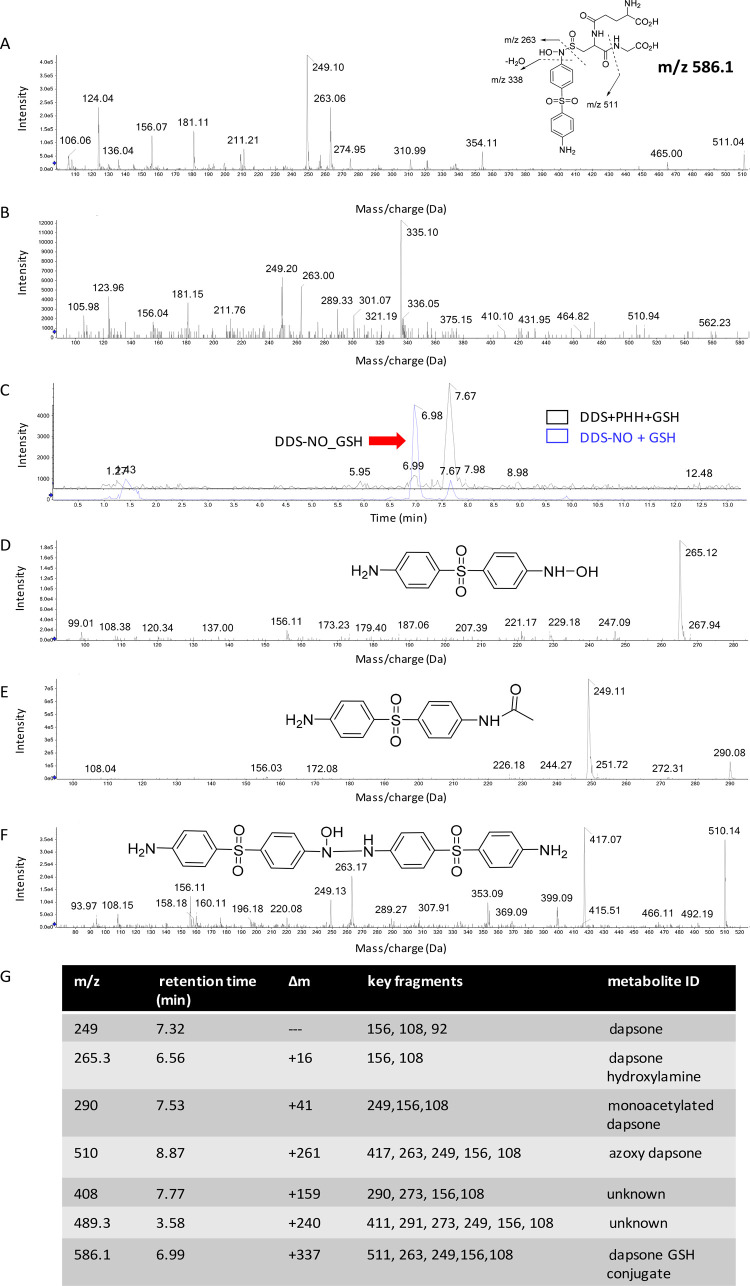
Mass spectrometric analysis of dapsone metabolites formed in the hepatocyte immune cell cocultures. (A) MS/MS spectra show that a sulfonamide glutathione adduct was formed by direct incubation of the synthetic nitroso dapsone with glutathione without hepatocytes. (B) Similar adduct was detected when dapsone was cultured with hepatocytes in the presence of glutathione. (C) Extracted ion chromatograms demonstrate that the glutathione adduct formed in the hepatocyte culture (black trace) has the same retention time as the synthetic adduct (blue trace). (D–F) MS/MS spectra show that additional dapsone metabolites including dapsone hydroxylamine (D), mono-acetyl dapsone (E), and azoxy dapsone (F) were also detected in hepatocyte immune cell cocultures. All dapsone metabolites detected in the hepatocyte immune cell cocultures are listed in (G).

Mass spectrometry was used to confirm that dapsone metabolites were formed in the hepatocyte immune cell cocultures. As nitroso dapsone is short-lived in culture, the nucleophile glutathione was used to trap nitroso dapsone. Nitroso dapsone was first cultured with GSH (1 mM) in the absence of hepatocytes and analyzed using LC–MS/MS. This positive control was performed to determine the retention time and MS fragmentation. As expected, a conjugate at retention time of 6.98 min with *m*/*z* 586.3 was detected, alongside abundant and characteristic fragmentation ions at *m*/*z* 263, 338, and 511, which are indicative of a sulfonamide glutathione adduct (Figure 7A). When dapsone was cultured with hepatocytes in the presence of glutathione, an adduct with similar MS/MS fragmentation and retention time was detected, indicating that the same dapsone glutathione adduct was obtained following incubation of dapsone with hepatocytes in the presence of glutathione (Figure 7B,C). This confirms the conversion of dapsone to nitroso dapsone under the coculture conditions. Dapsone, and additional dapsone metabolites, including dapsone hydroxylamine, mono-acetyl dapsone, and azoxy dapsone were also detected in the hepatocyte immune cell coculture supernatant (Figure 7D–F). Details of the dapsone metabolites generated including their *m*/*z* retention time, mass shift, fragmentation as well as assumed ID are listed in Figure 7G.

## Discussion

Drug hypersensitivity reactions remain an unpredictable, multifaceted burden on patients, healthcare providers, and the pharmaceutical industry.^29^ Several reactions are instigated by the formation of chemically reactive metabolites, which promote the formation of neoantigens for subsequent T-cell activation. These reactive species are only formed through bioactivation of parent compounds and insufficient amounts are detected in immune cultures by LC–MS/MS analysis or through T-cell activation assays with metabolite-responsive T-cells and the parent compound as a source of antigens. Thus, there is a need to develop novel assays where liver-derived metabolites can be generated in situ. Ultimately, there is a necessity to develop an assay which may bridge the existing gap between chemically reactive metabolite formation and subsequent T-cell activation within an in vitro model.

In this project, we utilized an in vitro hepatocyte metabolism system, which would hopefully generate a given metabolite in sufficient amounts to activate patient T-cells. To do this, we first selected the compound to study, namely, dapsone, which is associated with development of a hypersensitivity syndrome, driven at least in part by the formation of a chemically reactive nitroso metabolite.^23,30^ This metabolite has the propensity to activate T-cells; indeed, both CD4^+^ and CD8^+^ T-cell clones have been isolated and characterized from both healthy donors and patients with hypersensitivity.^7,15,23^ In our study, nitroso dapsone-responsive CD4+ T-cell clones from a hypersensitive patient were isolated and characterized in terms of drug metabolite specificity and pathways of drug-specific T-cell activation. To establish the assay conditions, it was of upmost importance to utilize clones displaying a response to nitroso dapsone but no response to parent drug. This was to ensure that all responses observed in our coculture assay were due to metabolite generation and not due to cross-reactivity with the parent drug. In total, nine nitroso dapsone-specific clones were characterized, each displaying proliferative responses and cytokine release when exposed to nitroso dapsone alone. These T-cell clones showed proliferative responses upon exposure to drug metabolite-pulsed antigen-presenting cells, while the response was attenuated when proliferation assays were conducted with fixed antigen-presenting cells or in the absence of antigen-presenting cells. Collectively, these data are indicative of the hapten pathway of drug presentation to T-cells with peptides derived from nitroso dapsone-modified protein associating with MHC class II molecules expressed on EBV-transformed B-cells for surface presentation to specific T-cells. Drug-pulsed antigen-presenting cells do not present drugs that stimulate T-cells via a pharmacological pathway as the weakly MHC-associated drug molecules are removed from MHC molecules expressed on the surface of B-cells through repeated washing steps.^7^ Fixation of B-cells with glutaraldehyde blocks antigen processing and hence the activation of T-cells with drug protein conjugates;^6,7^ however, preprocessed peptide antigens and pharmacological interacting drug antigens stimulate T-cell responses through direct binding to surface MHC molecules.^3,6,7^

Coculture experiments to assess the proliferative responses of nitroso dapsone-specific clones against in situ generated metabolites required the use of a metabolizing system. Although several options were available such as the HepaRG and HepG2 cell lines as well as subcellular liver fractions (S9, cytosol, and microsomes), for this study, we utilized primary human hepatocytes isolated from liver resections. Despite their limitations, hepatocytes are the gold standard in hepatotoxicity and metabolism studies.^31^ To ensure dapsone did not diminish the viability of hepatocytes, we treated hepatocytes with increasing concentrations of drug and assessed cell viability. No increase in cell death was observed with the parent compound at the concentrations used in the coculture assay. We next incorporated nitroso dapsone-specific clones in three plate formats to explore activation upon culture of dapsone with hepatocytes. In each format of the assay, antigen-presenting cells and T-cells were in one chamber, with hepatocytes in the other, with no physical interaction. First, the IdMOC plate was used as it offers a 96-well layout, with 16 separate chambers, thereby allowing for a multitude of experimental conditions. In subsequent experiments, hepatocytes and immune cells were cultured using multiwell semipermeable inserts or in-house 3D-printed coculture plates and the results compared. Although we were effectively able to culture our T-cell clones and hepatocytes within separate wells of the IdMOC plate, upon exposure to the parent compound, no significant T-cell activation was observed. This suggested that we may not be generating enough metabolite, particularly due to the small surface area and low number of hepatocytes included in the assay. Importantly, our negative control wells (containing all cell types but no drug) showed no increase in background counts, when compared with immune cells alone). Furthermore, the nitroso dapsone positive control wells indicated that T-cells were viable within the coculture and able to respond when presented with an appropriate antigen. In the next experiments, standard 24-well plates were employed with multiwell inserts, allowing for an increase in metabolite generating power (i.e., the number of hepatocytes) and culture with antigen-presenting cells and clones within the same overlaying medium. Upon culture of clones with hepatocytes in this layout, three tested clones were stimulated to proliferate in the presence of the parent compound (and nitroso dapsone control). Building on this assay, in-house 3D-printed coculture plates were generated, which allows for a culture system much like the IdMOC and multiwell inserts, increasing the metabolic power but requiring a smaller number of T-cell clones per well. This would allow for more experimental conditions, especially in scenarios where T-cells are available in low numbers. T-cell proliferative responses were similarly observed when clones were cultured with dapsone and the hepatocyte metabolite generating system. Collectively, these data indicate that nitroso dapsone-specific T-cell clones can be activated in the presence of the parent drug when metabolites are formed through hepatic metabolism. Comparison of the three coculture assays indicates that increasing hepatocyte numbers are critical for enhancing metabolic power within the system. Of note, all cocultures were conducted using the hepatocyte medium given the fragility of hepatocytes in other medium. Moving forward we are exploring whether it is possible to replace primary hepatocytes with cell lines expressing single drug metabolizing enzymes. This will have two advantages: (i) one can select the enzymes of interest for a given drug and (ii) the coculture can be conducted using a T-cell medium, which may enhance proliferation and the difference between negative control and drug-treated wells.

T-cell clones exposed to the positive control, nitroso dapsone, in coculture assays were found to proliferate to a lower extent when compared to the same clones cultured under classical conditions (i.e., clones, irradiated B-cells, and nitroso dapsone in the absence of hepatocytes in a 96-well round-bottomed plate). There are two possible explanations for this. First, the strength of response of clones to drugs always declines with time after several rounds of in vitro mitogen-driven expansion. Clones described in the paper were first assayed for phenotypes, cross-reactivity, and pathways of activation before being used in the coculture system; hence, the responses to the positive control were anticipated to be lower. Second, it is possible that the coculture conditions are not fully optimized when compared to a standard assay. Thus, in ongoing experiments, we are exploring experimental conditions such as immune cell numbers per well and duration of drug exposure in coculture plates before transfer of immune cells to the 96-well round-bottomed plates.

To ensure the observed T-cell responses were indeed a response to metabolite generation and not due to other cofounding factors, we utilized a nucleophilic chemical trapping agent, glutathione, to trap the nitroso metabolite formed. LC–MS/MS analysis revealed the presence of nitroso dapsone glutathione adducts in incubations containing the soluble metabolite but also importantly when dapsone was cultured in the complete experimental system containing hepatocytes and immune cells. Glutathione adducts were not in control cultures without hepatocytes or when glutathione was omitted from the assay. Based on these data, fresh clones are being generated to explore whether it is possible to block the hepatocyte-derived dapsone metabolite T-cell response through addition of reactive metabolite scavengers such as *N*-acetyl cysteine or glutathione.

Together, these results describe an in vitro coculture assay allowing in situ metabolite generation for subsequent T-cell activation studies. When developed further using dendritic cell T-cell cocultures, the assay could provide crucial insight into the importance of metabolite formation in naïve T-cell responses against novel drug structures, acting as a preclinical assay in the early phase of drug development, as well as providing insight into the importance of metabolite formation when hypersensitivity reactions are detected in clinical trials or when a drug enters the market. Several experimental steps are needed before using the coculture method to test new drug candidates. First, it is important to study additional compounds using allergic patient T-cell clones, where the T-cells have previously been shown to be activated with stable (atabecestat and allopurinol) or reactive metabolites (e.g., sulfamethoxazole). Second, to simplify the assay and obtain more reproducible results it will be important to generate and utilize liver-like cell lines transfected with single or multiple CYPs as metabolite generators. Then, it will then be possible to apply the revised coculture system to our previously described healthy volunteer dendritic cell, naïve T-cell priming assay.^32,33^ The same coculture assay could be used for diagnostic testing with hypersensitive patient PBMCs. The PBMC would be applied to the coculture assay in place or the transformed B-cells and T-cell clones prior to transfer to 96-well plates and assessment of proliferation and/or cytokine release.

## Abbreviations

PBMC: peripheral blood mononuclear cells
SI: stimulation index
EBV: Epstein–Barr virus
HLA: human leukocyte antigen
PI: pharmacological interaction
ELISpot: enzyme-linked immunospot
